# Energy landscape analysis of brain network dynamics in Alzheimer’s disease

**DOI:** 10.3389/fnagi.2024.1375091

**Published:** 2024-05-15

**Authors:** Le Xing, Zhitao Guo, Zhiying Long

**Affiliations:** ^1^The State Key Laboratory of Cognitive Neuroscience and Learning & IDG/McGovern Institute for Brain Research, Beijing Normal University, Beijing, China; ^2^School of Artificial Intelligence, Beijing Normal University, Beijing, China

**Keywords:** resting-state fMRI, energy landscape analysis, Alzheimer’s disease, brain dynamics, brain state

## Abstract

**Background:**

Alzheimer’s disease (AD) is a common neurodegenerative dementia, characterized by abnormal dynamic functional connectivity (DFC). Traditional DFC analysis, assuming linear brain dynamics, may neglect the complexity of the brain’s nonlinear interactions. Energy landscape analysis offers a holistic, nonlinear perspective to investigate brain network attractor dynamics, which was applied to resting-state fMRI data for AD in this study.

**Methods:**

This study utilized resting-state fMRI data from 60 individuals, comparing 30 Alzheimer’s patients with 30 controls, from the Alzheimer’s Disease Neuroimaging Initiative. Energy landscape analysis was applied to the data to characterize the aberrant brain network dynamics of AD patients.

**Results:**

The AD group stayed in the co-activation state for less time than the healthy control (HC) group, and a positive correlation was identified between the transition frequency of the co-activation state and behavior performance. Furthermore, the AD group showed a higher occurrence frequency and transition frequency of the cognitive control state and sensory integration state than the HC group. The transition between the two states was positively correlated with behavior performance.

**Conclusion:**

The results suggest that the co-activation state could be important to cognitive processing and that the AD group possibly raised cognitive ability by increasing the occurrence and transition between the impaired cognitive control and sensory integration states.

## 1 Introduction

Alzheimer’s disease (AD) is a neurodegenerative disease characterized by a progressive decline in memory and other cognitive abilities. At present, there is no curative treatment for AD, but medication can aid in controlling the symptoms and slowing the advancement of cognitive impairment. The functional magnetic resonance imaging (fMRI) technique has been widely applied to investigate the neural mechanism of Alzheimer’s disease (AD) and identify brain abnormalities specific to clinical behavior (Greicius et al., 2004; Buckner et al., 2009; Jagust, 2018).

Resting-state fMRI is a noninvasive imaging technique that indirectly measures synchronized blood oxygen level-dependent (BOLD) fluctuations in the brain, providing insights into the functional connectivity patterns of the brain (Biswal et al., 1995; Jagust, 2018). Among various resting brain networks, the default mode network (DMN) has been considered the core network reflecting AD pathological features. For AD patients, there was a significant decrease in the functional connectivity of the DMN (Greicius et al., 2004). In addition to the DMN, other brain networks, such as the executive control network (ECN) and salience network (SN), also showed changes induced by AD (Hahn et al., 2013; Badhwar et al., 2017). The alterations in functional connectivity may reflect cognitive dysfunction and compensatory responses to neurodegeneration damage in AD.

It should be noted that static functional connectivity analysis cannot capture the dynamic nature of the brain and fully reveal intricate temporal characteristics. Many previous studies have demonstrated that functional connectivity fluctuates dynamically with time (Chang and Glover, 2010). Dynamic functional connectivity (DFC) analysis has been used to distinguish functional states that vary between healthy controls and AD patients (Badhwar et al., 2017; Preti et al., 2017). Some studies have examined the dynamics of functional connectivity across diverse subnetworks, revealing aberrant dwell times specifically within memory-associated networks in AD brains (Jones et al., 2012; Filippi et al., 2019). Other studies demonstrated a gradual decline in whole-brain FC strengths that correlated with the increasing severity of cognitive impairment throughout the course of AD (Demirtaş et al., 2017; Pathak et al., 2022). The DFC analysis method used in previous studies assumes linearity of the brain dynamic system, while the dynamics of the brain are usually nonlinear (Hutchison et al., 2013; Preti et al., 2017). Thus, the dysfunction of the brain dynamic network in a complex and nonlinear brain system of AD remains to be elucidated.

Recently, energy landscape analysis based on statistical physics has been extensively applied in the analysis of brain dynamics during rest (Watanabe et al., 2014a; Ezaki et al., 2017, 2018; Kang et al., 2021; Klepl et al., 2022) and bi-stable visual perception tasks (Watanabe et al., 2014b). In contrast with the previous DFC method, energy landscape analysis has several advantages in exploring brain dynamics. First, energy landscape analysis can provide insights into the stability and robustness of brain dynamics because it reveals information about the attractor dynamics of a system. Second, energy landscape analysis is particularly useful in nonlinear systems and can provide a deeper understanding of brain dynamics. Third, energy landscape analysis provides a visual representation of the system, which can help to clarify and simplify complex brain systems.

Energy landscape analysis has been utilized to analyze abnormalities in brain dynamic dysfunction, including autism spectrum disorder (Watanabe and Rees, 2017), poststroke aphasia (Fan et al., 2022) and AD (Klepl et al., 2022; Li et al., 2023). For the AD patient, it was found that the dynamics of AD patients’ EEG were shown to be more constrained - with more local minima, less variation in basin size, and smaller basins (Klepl et al., 2022). Due to poor spatial resolution of EEG, the EEG study cannot reveal the dynamic changes of brain networks in AD patients. Li et al. (2023) applied the energy landscape analysis to the resting fMRI data of AD and investigate the dynamic changes of the DMN, the SN, and the ECN. The study revealed that the dynamics of patients with AD tend to be unstable, with an unusually high flexibility in switching between states (Li et al., 2023). However, Li’s study analyzes the DMN, SN and ECN separately by using the energy landscape method and ignored the interaction between the three networks. It has been demonstrated that several resting-state networks existed and interacted with each other in the resting fMRI data (Filippi et al., 2019; Puttaert et al., 2020; Cruzat et al., 2023). Therefore, it is essential to investigate the dynamic changes of the interactions between the resting networks by using the energy landscape analysis method to provide a more comprehensive understanding of the pathogenic mechanisms of Alzheimer’s disease.

**Figure 1:**
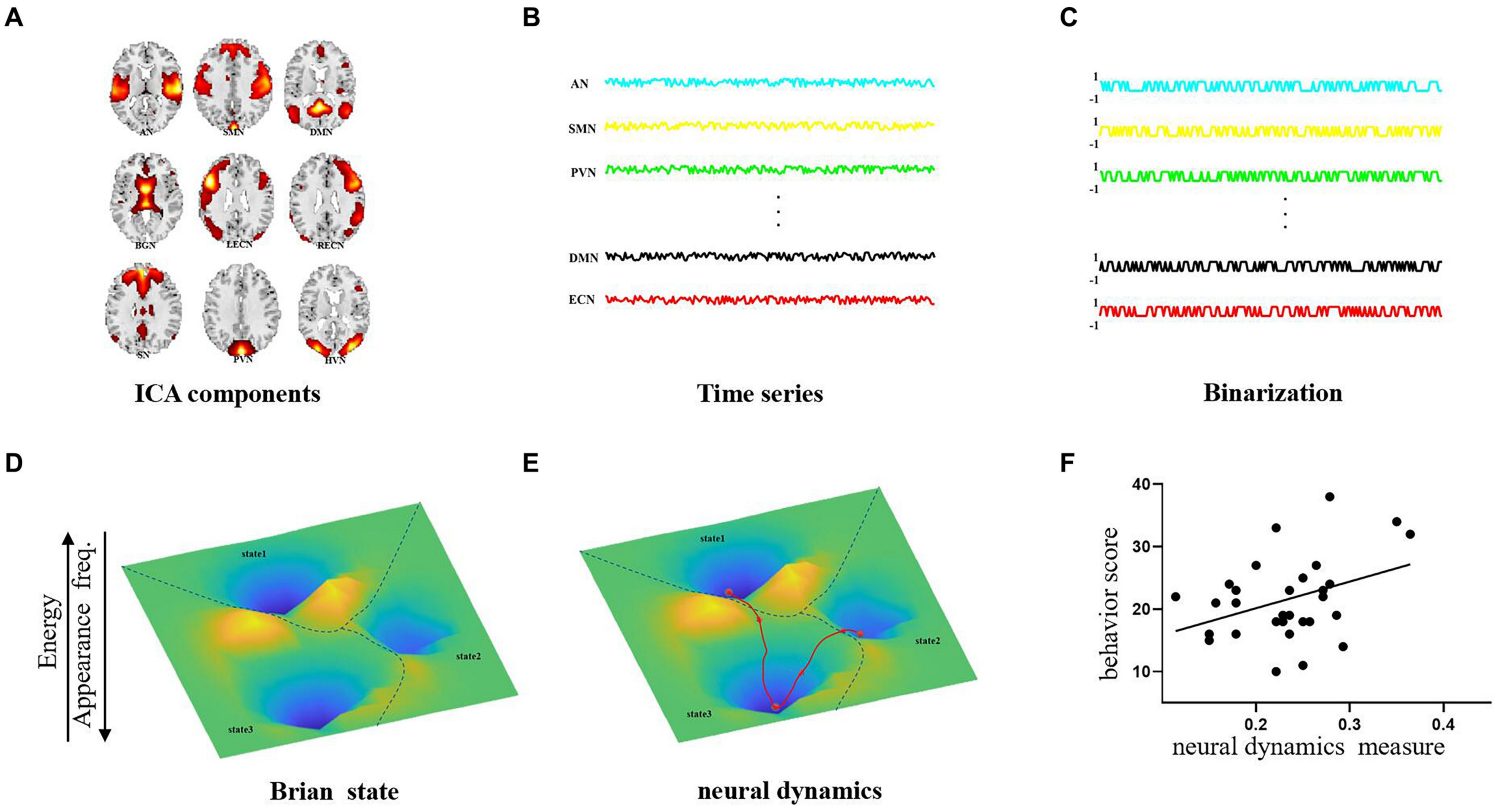
Pipeline of energy landscape analysis. **(A)** Functional brain network extraction by ICA. **(B)** The time series driving each network. **(C)** Binarized time series in **(B)**. **(D)** Identification of brain states in energy landscapes. **(E)** Extraction of the brain dynamic measures. **(F)** Relationship between brain dynamic measures and behavior scores.

This study aimed at revealing the abnormal dynamics of AD patient by treating all the resting-state networks as a whole activity pattern and examining the dynamic changes of the interactions between the resting networks collectively. In this study, we applied independent component analysis (ICA) to extract 9 resting fMRI networks and utilized the pairwise maximum entropy model to construct the energy landscape by using time series driving the 9 resting networks from 30 Alzheimer’s patients and 30 normal individuals. The maximum entropy model (MEM), as part of our energy landscape analysis, is used to estimate state energy distributions. We chose MEM due to its established effectiveness in deriving reliable and unbiased statistical inferences from limited datasets, with minimal prior assumptions (Jaynes, 1957; Schneidman et al., 2006). In the energy landscape analysis, the probability distribution of the brain states follows the Boltzmann distribution (the more frequently the state occurs, the lower its energy is). Our analysis methodology is illustrated schematically in Figure 1. The brain’s energy landscape comprises several valleys with local minima (referred to as “stable states” or “attractors”). The local minima have energy lower than their neighbours in the valley. The major brain activity patterns are defined as those that are frequently visited and situated in the lowest points of the energy landscape. We then assessed the ease of dynamic transitions by measuring the frequency of visits to these major activity patterns. The fMRI results revealed that AD symptoms could lead to an increased transition frequency between the cognitive control state and the sensory integration state as well as the occurrence frequency of the two states, which suggests that AD patients possibly used the compensatory mechanism to improve cognitive ability by increasing the transition frequency between the cognitive control and sensory integration states.

## 2 Materials and methods

### 2.1 Subjects

The dataset was from the Alzheimer’s Disease Neuroimaging Initiative (ADNI) database.^1^ The ADNI was established in 2003 as a public–private partnership led by Principal Investigator Michael W. Weiner, MD. The ADNI is a longitudinal database that aims to develop biomarkers for the early detection and tracking of AD progression. It collects clinical, imaging, genetic, and bio-specimen data related to AD and cognitive impairments. A total of 60 subjects, including 30 patients diagnosed with Alzheimer’s disease and 30 controls, were used in this study. Ethical approval was granted by the local ethical committees of all involved sites.

According to the protocols, AD was diagnosed based on a combination of clinical and cognitive assessments, along with biomarker data. The criteria used for AD diagnosis were primarily based on the National Institute on Aging-Alzheimer’s Association (NIA-AA) guidelines for the diagnosis of Alzheimer’s disease. Participants underwent a comprehensive battery of neuropsychological tests evaluating various cognitive domains, including memory, language, attention, executive function, and visuospatial skills. Key tests included the Mini-Mental State Examination (MMSE), Rey Auditory Verbal Learning Test (RAVLT), Montreal Cognitive Assessment (MoCA), Functional Activities Questionnaire (FAQ) and others. The sample descriptions are presented in Table 1. Details on Alzheimer’s Disease Stages can be found in Supplementary Table S1.

**Table 1:** Demographic data.

	HC	AD	*t* value	*p*-value
Age	74.69 ± 6.3	72.88 ± 6.3	1.08	0.284
Male/female	14/16	14/16		
MMSE	28.7 ± 1.3	22.5 ± 2.5	12.01	<0.001
MoCA	25.3 ± 1.9	16.1 ± 5.1	9.20	<0.001
RAVLT	43.03 ± 10.3	21.53 ± 6.6	9.58	<0.001
FAQ	0.06 ± 0.25	15.4 ± 7.64	−11.23	<0.001

### 2.2 Image acquisition

All participants underwent MRI scanning using a 3 T Philips MRI scanner to acquire resting-state functional images. The scanning protocol employed an echo-planar imaging (EPI) sequence with the following acquisition parameters: 140 volumes, repetition time (TR) = 3,000 ms, echo time (TE) = 30 ms, flip angle = 80°, number of slices = 48, slice thickness = 3.3 mm, spatial resolution = 3 × 3 × 3 mm^3^, and matrix size = 64 × 64. The original image files from this study are accessible to the broader scientific community for further analysis and research purposes. Additional information regarding the fMRI images can be found on the ADNI homepage.^2^

### 2.3 Data preprocessing

The present study utilized the Data Processing Assistant for Resting-State fMRI (DPARSF) software (Yan et al., 2016)^3^ to perform preprocessing steps on the rs-fMRI data. Specifically, each subject’s functional images underwent slice-timing correction, motion correction, and spatial normalization to the Montreal Neurological Institute (MNI) space. The resulting images were resliced into a voxel size of 3 × 3 × 3 mm and underwent spatial smoothing (Gaussian kernel with a full width at half maximum of 6 mm). Subsequently, linear detrending was applied, and the images were bandpass filtered (0.01–0.1 Hz).

### 2.4 Resting brain network extraction by ICA

Group independent component analysis (group-ICA) was performed to identify functional networks of the resting state. All preprocessed fMRI data were subjected to analysis using the GIFT toolbox.^4^ The optimal number of independent components (ICs) was estimated by the MDL criteria and was set to 30. The Component Labelling toolbox in GIFT was used to label the resulting independent components. Each component was identified according to its correlation with the resting-state network mask (Shirer et al., 2012). We finally selected 9 components (i.e., brain networks) relevant to AD according to a previous study (Badhwar et al., 2017): auditory network (AN), basal ganglia network (BGN), left executive control network (LECN), right executive control network (RECN), DMN, sensorimotor network (SMN), primary visual network (PVN), higher visual network (HVN), and SN.

### 2.5 Fitting of the pairwise MEM

To conduct energy landscape analysis, we applied the pairwise maximum entropy model (MEM) to the time series of the 9 brain networks in the same manner as in previous studies (Watanabe et al., 2014a; Watanabe and Rees, 2017; Ezaki et al., 2018; Fan et al., 2022). The pairwise MEM and the model fitting procedures are briefly described as follows.

For each group, the time series corresponding to N brain networks of S subjects were concatenated, and the data matrix Z_N╳ST_= $[Z_{N\times T}^{1}Z_{N\times T}^{2}\ldots Z_{N\times T}^{S}$ ] was obtained. T is the number of time points in the time series. In this study, N was equal to 9, T was equal to 140 and S was equal to 30. The matrix $Z_{N\times T}^{i}$ represents the time series of N networks for the *i*th subject. The nine mean values $m={\left[m_{1},m_{2},\ldots ,m_{N}\right]}^{T}$ that represent the average network activity of the nine brain networks were calculated by averaging Z across the columns. The data matrix Z was binarized based on the threshold of the mean values m. If Z(i,j) > m_i_, Z(i,j) = 1; otherwise, Z(i,j) = −1. A value of 1 represented the active state, while −1 represented the inactive state. Each column of Z represented the activity pattern of N networks at each time point. The total number of possible activity patterns amounted to 2^N^. $V_{k}=\left[\sigma_{1},\sigma_{2},\ldots ,\sigma_{N}\right]$ represents the *k*th activity pattern of all 2^N^ possible activity patterns, where $\sigma_{i}$ represents a binary activity of network *i*.

According to the principle of maximum entropy, the probability distribution $P\left(V_{k}\right)$ of the network activity pattern V_k_ should follow the Boltzmann distribution when the mean network activity and the mean pairwise interaction are constrained by the empirical data (see Eqs 1, 2).


(1) $$P\left(V_{k}\right)=e^{-E\left(V_{k}\right)}/\overset{2^{N}}{\underset{i-1}{\sum }}e^{-E\left(V_{k}\right)}$$

(2) $$E\left(V_{k}\right)=-\overset{N}{\underset{i-1}{\sum }}h_{i}\sigma_{i}\left(V_{k}\right)-\frac{1}{2}\overset{N}{\underset{i-1}{\sum }}\overset{N}{\underset{j-1,j\neq i}{\sum }}J_{ij}\sigma_{i}\left(V_{k}\right)\sigma_{j}\left(V_{k}\right)$$

Here, E(V_k_) is the energy of activity pattern V_k_, $\sigma_{i}\left(V_{k}\right)$ represents the binarized activity of network *i* in activity pattern $V_{k}$ , $h_{i}$ represents the activation tendency (baseline activity) of network *i* and $J_{ij}$ indicates a pairwise interaction between networks *i* and *j*. No interaction exists between networks i and j for $J_{ij}=0$ while interaction exists for $J_{ij}\neq 0$ . It is important to note that the energy *E* does not represent biological energy. Rather, it serves as a statistical indicator of the likelihood of occurrence of each brain activity pattern. Brain activity patterns with lower energy values tend to occur more frequently.

The model-based mean network activity ${\langle \sigma_{i}\rangle }_{m}=\Sigma_{k=1}^{2^{N}}\sigma_{i}\left(V_{k}\right)P\left(V_{k}\right)$ and model-based mean pairwise interaction ${\langle \sigma_{i}\sigma_{j}\rangle }_{m}=\Sigma_{k=1}^{2^{N}}\sigma_{i}\left(V_{l}\right)\sigma_{j}\left(V_{k}\right)P\left(V_{k}\right)$ were calculated using $P\left(V_{k}\right)$ in Eq. 1. The empirical mean network activity $\langle \sigma_{i}\rangle =\frac{1}{T}\overset{T}{\underset{t=1}{\sum }}\sigma_{i}^{t}$ and empirical mean pairwise interaction $\langle \sigma_{i}\sigma_{j}\rangle =\frac{1}{T}\overset{T}{\underset{t=1}{\sum }}\sigma_{i}^{t}\sigma_{j}^{t}$ were estimated from the empirical data, where $\sigma_{i}^{t}$ represented a binary activity of network i at time t. The parameters $h_{i}$ and $J_{ij}$ were iteratively adjusted by using a gradient ascent algorithm until ${\langle \sigma_{i}\rangle }_{m}$ and ${\langle \sigma_{i}\sigma_{j}\rangle }_{m}$ were approximately equal to the empirically obtained $\langle \sigma_{i}\rangle$ and $\langle \sigma_{i}\sigma_{j}\rangle$ for each group.

### 2.6 Accuracy of fit

According to previous studies (Watanabe et al., 2014a; Watanabe and Rees, 2017), the accuracy measure R was calculated by Eq. 3 to evaluate the goodness of fit of the pairwise MEM to the fMRI data of each group.


(3) $$R=\frac{D_{1-}D_{2}}{D_{1}}$$

(4) $$D_{1}=\overset{2^{N}}{\underset{k=1}{\sum }}p_{empirical}\left(V_{k}\right)\log_{2}\;\frac{p_{empirical}\left(V_{k}\right)}{p_{independent}\left(V_{k}\right)}$$

(5) $$D_{2}=\overset{2^{N}}{\underset{k=1}{\sum }}p_{empirical}\left(V_{k}\right)\log_{2}\;\frac{p_{empirical}\left(V_{k}\right)}{p\left(V_{k}\right)}$$

where $D_{1}$ represents the Kullback–Leibler divergence between the MEM with $J_{ij}=0$ and the empirical data, as calculated using Eq. 4. D_2_ represents the Kullback–Leibler divergence between the MEM with $J_{ij}\neq 0$ and the empirical data, was calculated using by Eq. 5. The accuracy measure R ranges from 0 to 1. MEM perfectly reproduces the empirical distribution of activity patterns for R = 1 and the pairwise interactions have no contribution to the MEM model fit for R = 0.

Additionally, the Pearson correlation coefficient between the appearance probability derived from the pairwise MEM and the empirical appearance probability was calculated. The greater the Pearson correlation coefficient was, the more accurately the maximum entropy model could explain the empirical data.

### 2.7 Construction of energy landscapes

To characterize the resting dynamics of the brain system, an energy landscape analysis was conducted for each group according to the procedure described in previous works (Watanabe et al., 2014a; Watanabe and Rees, 2017).

A network of activity patterns was constructed by setting each activity pattern as a network node. Two nodes were defined as adjacent if the two activity patterns were the same across all brain networks except one. Therefore, an activity pattern $V_{k}$ was adjacent to N activity patterns. The network of activity patterns with their node energy E $\left(V_{k}\right)$ was defined as the energy landscape. In the energy landscape, the local energy minima (attractors) were the nodes with energy values smaller than those of their N adjacent nodes.

**Figure 2:**
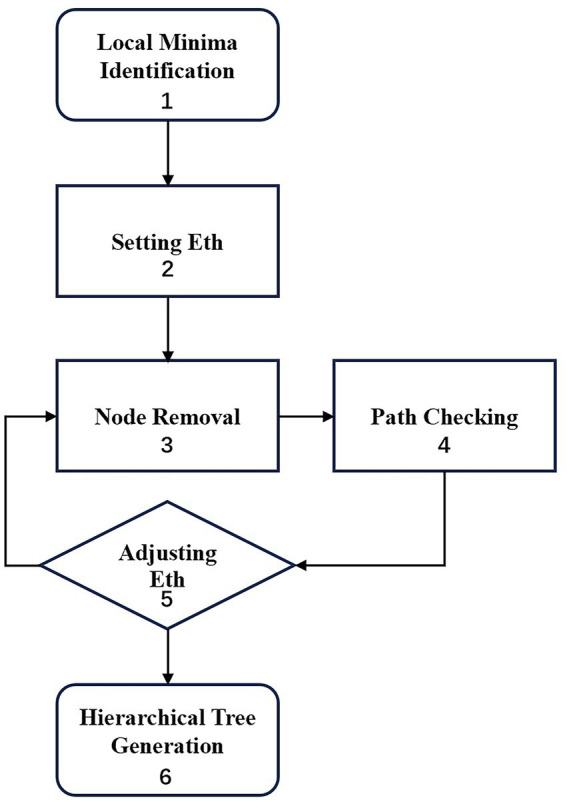
Work flow of dysconnectivity graph.

A dysconnectivity graph (Becker and Karplus, 1997) was constructed according to the following six steps, as depicted in the flowchart in Figure 2. First, all the local energy minima were achieved by thoroughly searching the energy landscape. Second, the energy threshold E_th_, was set to the highest energy value present among all 2^N^ nodes. Third, the nodes with energy values equal to or larger than E_th_ were removed. Fourth, a connectivity check was performed to ensure that each pair of local minima was connected via a path within the reduced network. Fifth, E_th_ was adjusted by the subsequent largest energy value. The third, fourth and fifth steps were repeated until each local minimum was isolated in a reduced network. Finally, a hierarchical tree was generated. The terminal leaves represented the local minima, and the internal nodes indicated the branching points of different local minima. The leaves’ vertical positions reflected their energy values.

### 2.8 Estimation of the sizes of dominant brain states

The basin sizes of the detected local minima were used to measure the dominance of each local minimum. A starting node i was selected from the 2^N^ nodes. If there was any neighbouring node that had lower energy than node i, node i was moved to its neighbouring node with the lowest energy value. If no such neighbouring node existed, node i was a local minimum, and no movement occurred. This procedure was repeated until a local minimum was obtained. The starting node i was defined as an element of the basin of the local minimum that was reached by the starting node i. This process was applied to all 2^N^ nodes repeatedly. All the brain activity patterns that belonged to the basin constituted the basin of a local minimum. Consequently, the basin size was defined as the fraction of the number of brain activity patterns belonging to the basin.

In this study, six local minima were grouped into three brain states according to the hierarchical structures of these minima (local minima 1, 2 and 4 for State 1; local minima 3 and 5 for State 2 and local minimum 3 for State 3; Figure 1C). The size of each brain state was defined as the summation of the basin sizes of all the local minima that belonged to the brain state.

### 2.9 Dynamic measures of brain states

According to the definition of the three brain states, the activity pattern at each time point was classified as one of the three brain states for each participant. Seven dynamic measures, including the appearance frequency, mean duration, mean energy, transition frequency, direct transition frequency, indirect transition frequency and transition in/out frequency, were calculated from the empirical data of each subject. For each participant, the appearance frequency of a state was calculated as the ratio of the occurrence number of the state to the total number of all states observed. The mean dwell time of a state was measured as the average number of consecutive occurrences of the state. The mean energy was calculated by averaging the energy levels of states across all time points. The state transition frequency from state A to B was measured as the ratio of the transition number from state A to B to the total transition number between states. Specifically, the state transition was further classified into direct transition and indirect transition. Indirect transitions involved intermediate states between the initial and target states. The transition in/out frequency was defined as the transition frequency at which a particular state transitioned to or from other states. Two-sample t tests with a Bernoulli correction were applied to all dynamic measures to evaluate the differences between the HC and AD groups.

### 2.10 Numerical simulations

According to the energy landscape estimated for each group (HC/AD), the movement of the brain activity patterns was numerically simulated using a Markov chain Monte Carlo method with Metropolis-Hasting’s algorithm (Metropolis et al., 1953; Hastings, 1970). In this method, any brain activity pattern $V_{i}$ was only allowed to move to its neighbour pattern $V_{j}$ that was selected from all *N* neighbours with a uniform probability of 1/*N*. The probability of transition from $V_{i}$ to $V_{j}$ was $P_{ij}=\min\left[1,e^{E\left(V_{i}\right)-E\left(V_{j}\right)}\right]$ . For each group, we repeated the random walk of 10^5^ steps with a randomly selected initial pattern $V_{k}$ so that the simulated data could fully describe the transition of the brain activity pattern. Using this numerical simulation, the mean duration, mean energy and transition probability of the brain states were calculated to characterize the brain dynamics. The differences in brain dynamics between the AD and HC groups were assessed through the utilization of chi-square (χ2) tests and *post hoc* residual tests.

### 2.11 Associations between brain dynamics and behaviors

We further explored the relationships between brain dynamics and behavior performance. The analysis focused on the DFC measures that showed significant intergroup differences. The Pearson correlations between behavior performances and the brain dynamic measures were calculated for the two groups separately.

## 3 Results

### 3.1 Accuracy of model fitting

**Figure 3:**
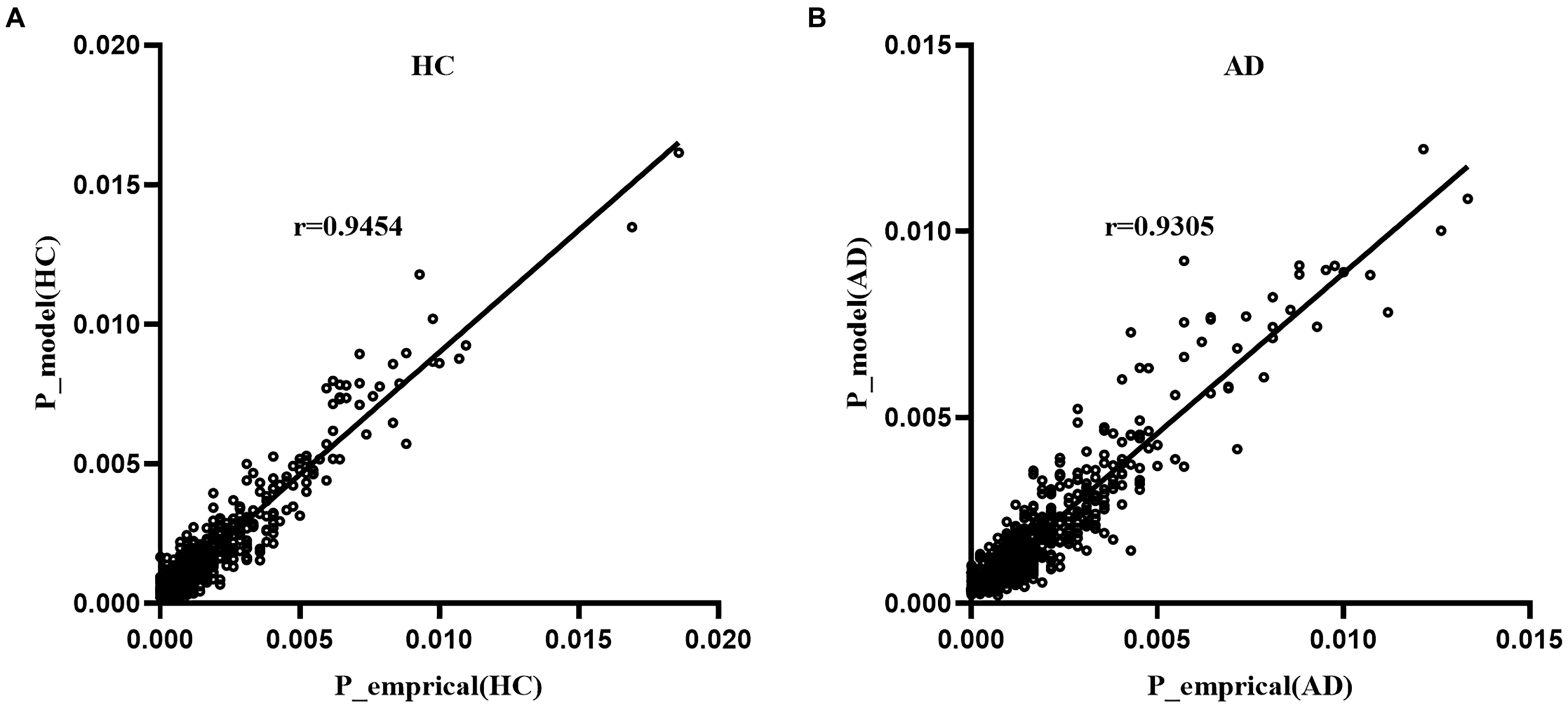
Fitting of the pairwise MEM for the AD group **(A)** and HC group **(B)**.

Figure 3 displays the fitting results of the pairwise MEM for the empirical data of the AD and HC groups. The results indicated that the MEM could accurately predict the empirical data for both the AD and HC groups (accuracy: R_AD_ = 0.822, R_HC_ = 0.836). Moreover, the appearance probability derived from the pairwise MEM was highly correlated with the empirical appearance probability for both groups (r_AD=_0.930_,_ *p* < 0.001; r_HC=_0.945, *p* < 0.001).

### 3.2 Energy landscapes and basin size

**Figure 4:**
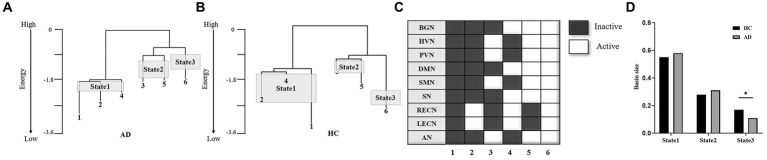
Comparison of energy landscape structures. **(A,B)** Hierarchal structure of the energy landscape for the AD **(A)** and HC **(B)** groups. **(C)** The six local minima of the landscapes of the two groups. **(D)** Basin sizes of the three brain states for the two groups. *p < 0.05.

The hierarchal structures of the two groups’ energy landscapes are shown in Figures 4A,B. The energy landscapes of the AD and HC groups showed a similar hierarchal structure with the same six local minima (Figure 4C). The six local minima were grouped into three brain states (local minima 1, 2 and 4 for State 1; local minima 3 and 5 for State 2; local minimum 3 for State 3). Figure 4D shows the sizes of the three brain states of the two groups. The distributions of the brain states were significantly different between the two groups (χ^2^ = 7.95, *p* < 0.05 in a χ^2^-test). For State 3, the state size of the AD group was significantly smaller than that of the HC group (*p* < 0.05 in a *post hoc* residual test).

### 3.3 Characterization of brain dynamics

**Figure 5:**
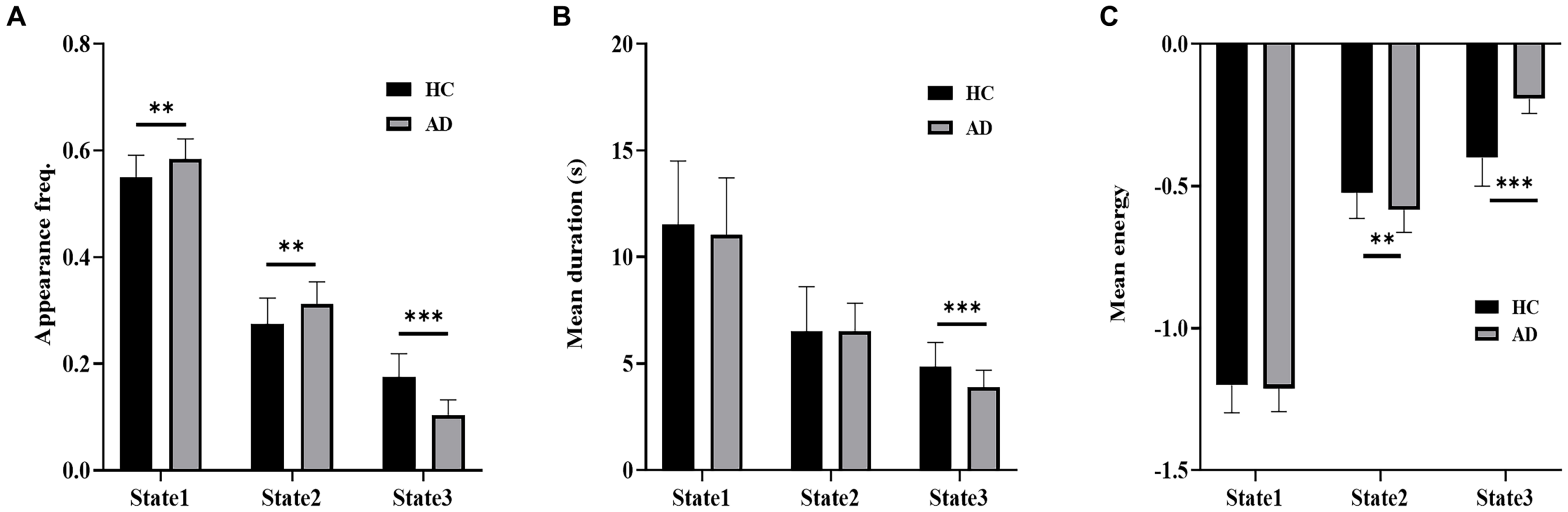
Dynamic properties of brain states. **(A)** Appearance frequency of the three brain states for the HC and AD groups. **(B)** Mean duration of the three brain states for the HC and AD groups. **(C)** Mean energy of the three brain states for the HC and AD groups. ^*^p < 0.05, ^**^p < 10^−2^, ^***^p < 10^−3^.

Figure 5 shows a comparison of the appearance frequency, mean duration and mean energy of the three states in the empirical data. In contrast to the HC group, the AD group displayed a significantly higher appearance frequency in State 1 (t = 3.33, *p* < 10^−2^, P_Bonferroni_ < 0.05) and State 2 (*t* = 3.14, *p* < 10^−2^, P_Bonferroni_ < 0.05) and a significantly lower appearance frequency in State 3 (*t* = 7.46, *p* < 10^−3^, P_Bonferroni_ < 0.05). Moreover, the HC group exhibited a significantly longer mean duration of State 3 than the AD group (t = 3.88, *p* < 10^−3^, P_Bonferroni_ < 0.05). State 2 showed significantly higher mean energy (*t* = 2.77, *p* < 10^−2^, P_Bonferroni_ < 0.05), and State 3 showed significantly lower mean energy in the HC group versus the AD group (*t* = 10.06, *p* < 10^−3^, P_Bonferroni_ < 0.05).

**Figure 6:**
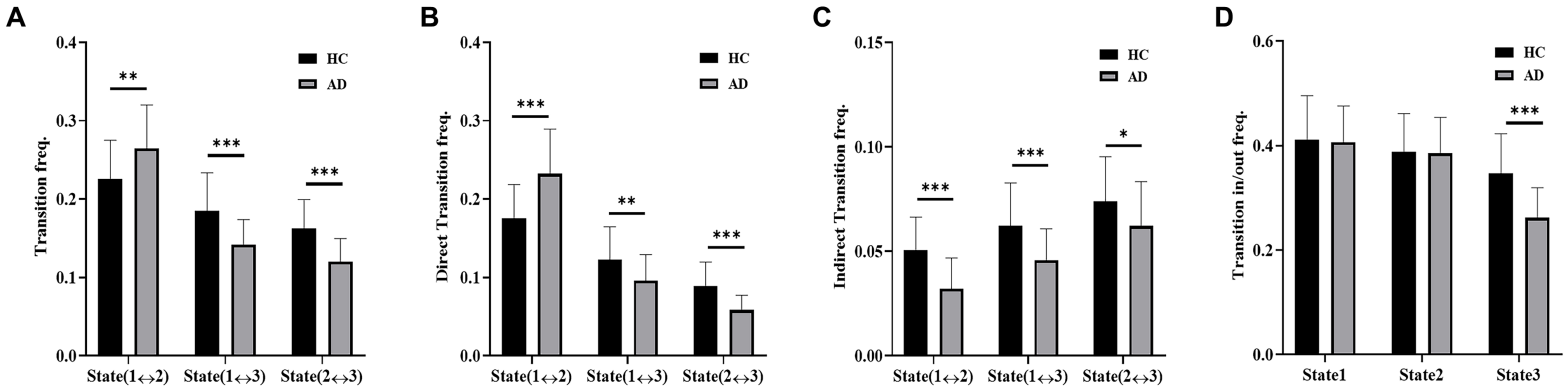
Dynamic transition of states. **(A)** Transition frequency of the AD and HC groups. **(B)** Direct transition frequency of the AD and HC groups. **(C)** Indirect transition frequency of the AD and HC groups. **(D)** Transition in/out frequency of the AD and HC groups.

The transition frequencies of the three states in the empirical data of the two groups are presented in Figure 6. The transition frequency, including direct transition and indirect transition, between States 1 and 2 was significantly reduced in the HC group compared to the AD group (*t* = 2.83, *p* < 10^−2^, P_Bonferroni_ < 0.05). Compared with the AD group, the HC group showed a significantly higher transition frequency between States 1 and 3 (*t* = 4.03, *p* < 10^−3^, P_Bonferroni_ < 0.05) and between States 2 and 3 (*t* = 4.85, *p* < 10^−3^, P _Bonferroni_ < 0.05). For the direct transition frequency, the HC group showed a significantly lower frequency between States 1 and 2 (*t* = 4.35, *p* < 10^−3^, P_Bonferroni_ < 0.05) and a significantly higher frequency between States 1 and 3 (*t* = 2.73, *p* < 10^−2^, P_Bonferroni_ < 0.05) and between States 2 and 3 (*t* = 4.55, *p* < 10^−2^, P_Bonferroni_ < 0.05) compared with the AD group. For the indirect transition frequency, the HC group showed a significantly higher frequency between States 1 and 2 (*t* = 4.73, *p* < 10^−3^, P_Bonferroni_ < 0.05), between States 1 and 3 (*t* = 3.53, *p* < 10^−3^, P_Bonferroni_ < 0.05), and between States 2 and 3 (*t* = 2.11, *p* < 10^−2^, P_Bonferroni_ < 0.05) than the AD group. Moreover, the transition in/out frequency of State 3 was significantly higher for the HC group than for the AD group (*t* = 4.91, *p* < 10^−3^, P_Bonferroni_ < 0.05).

### 3.4 Association between brain dynamics and cognitive ability

**Figure 7:**
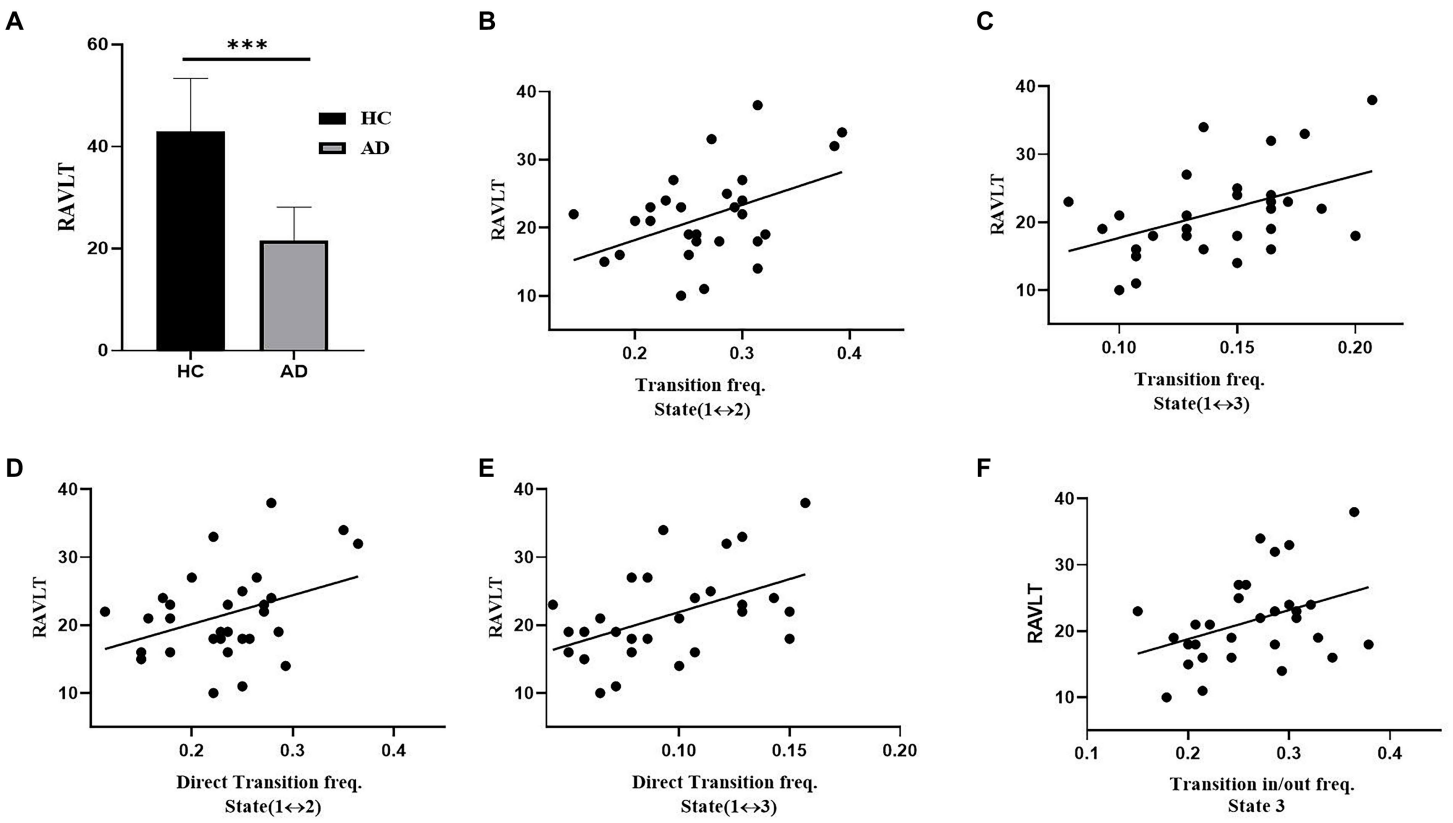
Association between transition rates and cognitive ability (RAVLT) in the AD group. **(A)** RAVLT scores of the HC and AD groups. **(B)** The relation between RAVLT and the transition frequency of States 1 and 2. **(C)** The relation between RAVLT and the transition frequency of States 1 and 3. **(D)** The relation between RAVLT and the direct transition frequency of States 1 and 2. **(E)** The relation between RAVLT and the direct transition frequency between States 1 and 3. **(F)** The relation between RAVLT and the transition in/out frequency of State 3.

Figure 7 illustrates the relationships that had a significant correlation between neuropsychological assessments and the measurements of brain dynamics. For the RAVLT, the average score of AD patients was significantly lower than that of HCs (*t* = 9.58, *p* < 10^−3^, P_Bonferroni_ < 0.05). For the AD group, the RAVLT score showed a significant positive correlation with the transition frequency between States 1 and 2 (*r* = 0.55, *p* < 0.05), the transition frequency between States 1 and 3 (*r* = 0.45, *p* < 0.05), the direct transition frequency between States 1 and 2 (*r* = 0.43, *p* < 0.05), the direct transition frequency between States 1 and 3 (*r* = 0.45, *p* < 0.05), and the transition in/out frequency of State 3 (*r* = 0.45, *p* < 0.05).

### 3.5 Numerical simulation results

**Figure 8:**
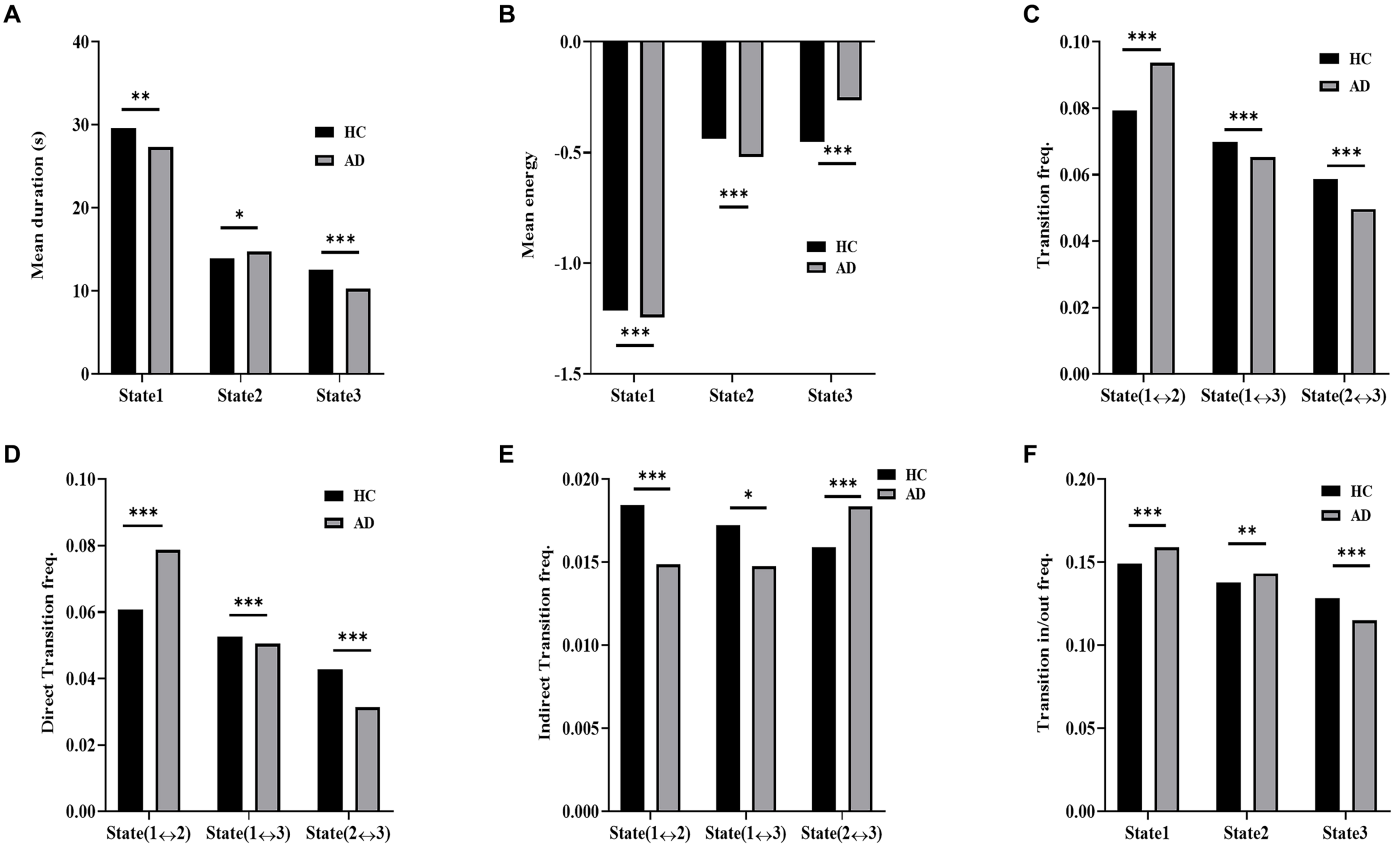
Dynamic measurement comparison of simulated data. **(A)** Mean duration of HC and AD. **(B)** Mean energy of HC and AD. **(C)** Transition frequency of AD and HC. **(D)** Direct transition frequency of AD and HC. **(E)** Indirect transition frequency of AD and HC. **(F)** Transition in/out frequency of AD and HC.

The dynamic measurements of the three states in the simulated data of the AD and HC groups are displayed in Figure 8. The distributions of the mean duration of each state exhibited significant differences between the two groups (χ^2^ = 106.9, *p* < 10^−3^ in a χ^2^-test). In contrast to the AD group, the HC group showed a significantly longer mean duration for State 1 (*p* < 10^−3^ in a *post hoc* residual test) and State 3 (*p* < 10^−4^ in a *post hoc* residual test) and a significantly shorter duration for State 2 (*p* < 0.05 in a *post hoc* residual test). The distribution of the mean energy of states displayed a significant difference between the HC and AD groups (χ^2^ = 1272.17, *p* < 10^−3^ in a χ^2^-test). The mean energy of the AD group was significantly larger for State 1 (*p* < 10^−4^ in a *post hoc* residual test) and State 2 (*p* < 10^−4^ in a *post hoc* residual test; Figure 8B) and significantly lower for State 3 compared to that of the HC group (*p* < 10^−4^ in a *post hoc* residual test).

Moreover, significant intergroup differences were observed in the distributions of state transition probabilities, including transition probability (χ^2^ = 208.9, *p* < 10^−3^ in a χ^2^-test), direct transition probability (χ^2^ = 404.4, *p* < 10^−3^ in a χ^2^-test), indirect transition probability (χ^2^ = 61.7, *p* < 10^−3^ in a χ^2^-test), and transition in/out probability (χ^2^ = 116.3, *p* < 10^−3^ in a χ^2^-test) in the simulated data. For both the transition frequency and direct transition frequency, the HC group exhibited a significantly higher frequency between States 1 and 3 (*p* < 10^−4^ in a *post hoc* residual test) and between States 2 and 3 (p < 10^−4^ in a *post hoc* residual test; Figure 8A) than the AD group, while the AD group had a significantly higher frequency between States 1 and 2 (*p* < 10^−4^ in a *post hoc* residual test) than the HC group. For indirection transitions, the frequency of the HC group was significantly higher between States 1 and 2 (*p* < 10^−4^ in a *post hoc* residual test) and between States 1 and 3 (*p* < 0.05 in a *post hoc* residual test) and significantly lower between States 2 and 3 (*p* < 10^−4^ in a *post hoc* residual test) compared to that of the AD group. For the transition in/out frequency, the HC group displayed a significantly lower frequency for State 1 (*p* < 10^−4^ in a *post hoc* residual test) and State 2 (*p* < 10^−3^ in a *post hoc* residual test) and a significantly higher frequency for State 3 in contrast to the AD group (*p* < 10^−4^ in a *post hoc* residual test).

## 4 Discussion

In the present study, we investigated the impact of AD on resting-state dynamics by using an energy landscape analysis. Both the AD and HC groups showed dynamic direct and indirect transitions among the cognitive control state (State 1), the sensory integration state (State 2) and the co-activation state (State 3). In contrast to the HC group, the AD group spent significantly less time in State 3, and State 1 and State 2 occurred significantly more frequently. Moreover, the AD group tended to switch directly between State 1 and State 2, while the HC group tended to transit in/out of State 3. In the AD group, the RAVLT score showed a positive correlation with the transition in/out frequency of State 3 and the transition frequency between State 1 and State 2. The results suggest that State 3 could play an important role in cognitive processing and that AD patients possibly use the compensatory mechanism to improve cognitive ability by increasing the transition frequency between State 1 and State 2.

### 4.1 Brain state

A similar hierarchal structure with the same six local minima was detected in the energy landscapes of the AD and HC groups. The first local minima with all 9 networks inactivated may represent an inactive state. The second local minimum with RECN and LECN activation possibly represents the cognitive control state (Joo et al., 2016). The third local minimum with HVN, PVN, SMN and AN activation possibly represents a sensory processing state that includes visual, auditory and motor perception (Hutchison et al., 2013). The fourth local minimum exhibits activation of the BGN, DMN, SN, RECN and LECN. The SN has been implicated in modulating the switch between the internally directed cognition of the DMN and the externally directed cognition of the RECN and LECN (Sridharan et al., 2008). Thus, the fourth local minimum may represent a switching state between internal and external cognition. The fifth local minimum exhibits activation of the BGN, HVN, PVN, DMN, SMN, SN and AN. Because the SN plays a role in the detection and integration of salient sensory stimuli (Downar et al., 2000), it may work with the HVN, PVN and AN to detect and integrate visual and auditory stimuli. The BGN was reported to be involved in motor control (Turner and Desmurget, 2010) and may work with the SMN to perform motor processing. Thus, the fifth local minimum may be relevant to the integration of various sensory stimuli and motor function. The sixth local minimum with all networks activated represents an activated state.

In the hierarchal structure of the energy landscape, local minima 1, 2 and 4 were on the left branch, and local minima 3 and 5 were on the same branch for the AD and HC groups. Local minima 1, 2 and 4 were grouped into State 1, which may be related to cognitive control. Local minima 3 and 5 were grouped into State 2, which may be related to the integration of sensory stimuli and motor processing. Local minimum 6 was assigned to State 3, which may be related to the co-activation state.

### 4.2 Dynamic property of brain states

In contrast to the HC group, the AD group showed a significantly lower mean energy in State 2 and a higher mean energy in State 3. Because subjects prefer to stay in a stable state with low energy, State 3 occurred less frequently and State 2 more frequently in the AD group than in the HC group (Figure 5A). In State 3, all 9 networks cooperated and showed co-activation. Many previous studies observed altered functional connectivity between brain regions in AD patients (Filippi et al., 2019). Because the altered functional connectivity pattern may impact the co-activation of the 9 networks, State 3 occurred less frequently in the AD group than in the HC group. This result may suggest that the 9 networks of the HC group tended to interact more closely with each other than those of the AD group. State 2 was relevant to sensory and motor processing. Previous evidence indicated that sensory and motor changes may precede the cognitive symptoms of AD by several years and may signify an increased risk of developing AD (Albers et al., 2015). Moreover, visual, auditory and motor dysfunctions are selectively observed in subgroups of AD patients (Albers et al., 2015; Weintraub et al., 2018). Therefore, the higher appearance frequency of State 2 in AD patients possibly provides a compensatory mechanism for sensory and motor impairment in AD (Brier et al., 2012). State 1 showed a significantly higher appearance frequency in the AD group than in the HC group. In State 1, the SN, CEN and DMN interacted closely to control cognitive processes. It has been reported that functional organizations of CEN and DMN were impaired in AD (Joo et al., 2016), and functional connectivity between the SN and the other two networks (CEN and DMN) was also altered in AD patients (He et al., 2014). Furthermore, a triple-network (SN, CEN and DMN) analysis of Alzheimer’s disease, focusing on the energy landscape of these networks, revealed that the dynamics of patients with AD tend to be unstable, suggesting fluctuations in network interactions (Li et al., 2023). An increased occupancy rate in State 1 of AD patients possibly suggests a potential brain compensatory mechanism for enhancing cognitive control and coordination.

In terms of the transition between states, AD patients switched directly between State 1 and State 2 more frequently and between State 3 and the other two states less frequently than HCs. The results indicated that the AD group liked to stay in States 1 and 2, whereas the HC group liked to stay in State 3, which was consistent with the results of appearance frequency. It was reported that transitions to co-activated states within brain networks became more challenging in the aging population (Ezaki et al., 2017), which is consistent with the finding that the transition to State 3 is suppressed in AD patients in this study. For the AD group, the increased transition frequency between State 1 and State 2 and the higher appearance frequency of State 1 and State 2 may serve as a compensatory strategy to offset cognitive impairment in sensory processing and cognitive control (Brier et al., 2012; Hillary et al., 2015).

### 4.3 Association between brain dynamics and cognitive ability

Associations between atypical brain dynamics and cognitive ability were observed in AD patients. These associations were also observed in other cognitive impairment patients (McKeith et al., 2017; Premi et al., 2019). For the AD group, the transition frequency between State 1 and State 2 showed a significantly positive correlation with the RAVLT score (Figure 7B). The RAVLT score is often used to evaluate verbal memory performance (Moradi et al., 2017). High RAVLT score represents high verbal memory performance. In contrast to HCs, AD patients had a significantly lower RAVLT score due to their memory impairment (Figure 7A). However, the AD group showed a significantly higher transition frequency between State 1 and State 2 than the HC group. Thus, the positive correlation between the transition frequency and RAVLT score further supported the compensatory mechanism in the AD group of enhancing the RAVLT score by increasing the transition frequency between State 1 and State 2. Moreover, the RAVLT score of the AD group was highly correlated with the transition in/out frequency of State 3 and the transition frequency between State 1 and State 3 (Figure 7F). In contrast to the HC group, the AD group showed a significantly lower transition in/out frequency of State 3 and transition frequency between State 1 and State 3. For the AD group, subjects with a higher transition of State 3 had less impairment of coactivation of all 9 networks, and subjects with a high RAVLT score had less cognitive impairment. Therefore, the AD group displayed a positive correlation between the transition frequency of State 3 and the RAVLT score, which may suggest that the high transition frequency of State 3 improves the RAVLT score.

## 5 Conclusion

In summary, this study explored the brain dynamics of AD patients by applying energy landscape analysis to resting-state fMRI data. Three stable brain states were identified from both the AD group and the HC group. The results revealed that cognitive impairments in AD primarily reduced the average dwell time in State 3 and the transition frequencies associated with State 3. The positive correlation between the transition in/out frequency of State 3 and RAVLT score of the AD group suggested that State 3 was important for cognitive processing. Furthermore, the increased transition frequency of State 1 and State 2 that was positively correlated with the RAVLT score suggests a compensatory mechanism of the AD group to raise cognitive ability by increasing the occurrence and transition between the two states with impaired cognitive function.

## Data availability statement

The original contributions presented in the study are included in the article/Supplementary material, further inquiries can be directed to the corresponding author.

## Ethics statement

The studies involving humans were approved by Alzheimer's Disease Neuroimaging Initiative. The studies were conducted in accordance with the local legislation and institutional requirements. The participants provided their written informed consent to participate in this study. Written informed consent was obtained from the individual(s) for the publication of any potentially identifiable images or data included in this article.

## Author contributions

LX: Conceptualization, Data curation, Formal analysis, Methodology, Software, Writing – original draft, Writing – review & editing. ZG: Data curation, Methodology, Software, Conceptualization, Writing – review & editing, Writing – original draft. ZL: Conceptualization, Funding acquisition, Investigation, Methodology, Writing – original draft, Writing – review & editing.

## Footnotes

^1^ http://adni.loni.usc.edu/

^2^ http://adni.loni.usc.edu/methods/mri-tool/mri-analysis/

^3^ http://rfmri.org/dpabi

^4^ mialab.mrn.org/software/gift/
