# Supplementary material for: Energy landscape analysis of brain network dynamics in Alzheimer’s disease
Source: Front Aging Neurosci. 2024 May 15;16:1375091. doi: 10.3389/fnagi.2024.1375091 (PMC11133694; doi:10.3389/fnagi.2024.1375091)
Supplement: Supplementary file 1 [file Table_1.DOCX]

Table S1: Alzheimer’s Disease Stages Information

| PTID | AGE | PTGENDER | MMSE | AD_Stage |
| --- | --- | --- | --- | --- |
| 002_S_5018 | 73.3 | Male | 23 | Early-stage |
| 006_S_4153 | 79.3 | Male | 22 | Early-stage |
| 006_S_4192 | 82.2 | Male | 19 | Middle-stage |
| 006_S_4546 | 71 | Male | 25 | Early-stage |
| 006_S_4867 | 74.4 | Male | 23 | Early-stage |
| 013_S_5071 | 76 | Male | 19 | Middle-stage |
| 018_S_4696 | 73 | Female | 20 | Middle-stage |
| 018_S_4733 | 75.3 | Male | 26 | Early-stage |
| 018_S_5240 | 62.7 | Female | 20 | Middle-stage |
| 019_S_4252 | 86.5 | Female | 22 | Early-stage |
| 019_S_4477 | 82.1 | Female | 21 | Early-stage |
| 019_S_4549 | 79 | Male | 21 | Early-stage |
| 019_S_5012 | 76.3 | Male | 25 | Early-stage |
| 019_S_5019 | 62.9 | Female | 21 | Early-stage |
| 031_S_4024 | 55.9 | Female | 25 | Early-stage |
| 053_S_5070 | 71.2 | Male | 26 | Early-stage |
| 053_S_5208 | 68.7 | Male | 26 | Early-stage |
| 100_S_5106 | 74.1 | Male | 20 | Middle-stage |
| 130_S_4589 | 75.1 | Female | 26 | Early-stage |
| 130_S_4641 | 73.6 | Female | 26 | Early-stage |
| 130_S_4660 | 77.2 | Female | 24 | Early-stage |
| 130_S_4730 | 81.1 | Female | 21 | Early-stage |
| 130_S_4971 | 76.4 | Male | 21 | Early-stage |
| 130_S_4982 | 58.2 | Female | 21 | Early-stage |
| 130_S_4984 | 73.1 | Female | 20 | Middle-stage |
| 130_S_4990 | 75.1 | Female | 25 | Early-stage |
| 130_S_4997 | 60.6 | Female | 19 | Middle-stage |
| 130_S_5006 | 68.2 | Female | 21 | Early-stage |
| 130_S_5059 | 72.1 | Male | 21 | Early-stage |
| 136_S_4993 | 71.8 | Female | 26 | Early-stage |
